# Integrative single-cell and bulk RNA-seq analyses identify CD4^+^ T-cell subpopulation infiltration and biomarkers of regulatory T cells involved in mediating the progression of atherosclerotic plaque

**DOI:** 10.3389/fimmu.2024.1528475

**Published:** 2025-01-17

**Authors:** Yifeng Zhang, Shuxian Lu, Liang Qiu, Manman Qin, Dan Shan, Lianhua Xie, Yao Yi, Jun Yu

**Affiliations:** ^1^ Discipline of Chinese and Western Integrative Medicine, Jiangxi University of Chinese Medicine, Nanchang, China; ^2^ Centre for Translational Medicine, Jiangxi University of Chinese Medicine, Nanchang, China; ^3^ Department of Cardiovascular Sciences, Centre for Metabolic Disease Research, Lewis Katz School of Medicine, Temple University, Philadelphia, PA, United States; ^4^ Institute of Obstetrics and Gynecology, Jiangxi University of Chinese Medicine, Nanchang, China

**Keywords:** atherosclerosis, biomarker, immune cell infiltration, regulatory T cells, single-cell RNA-seq

## Abstract

**Background:**

Atherosclerosis (AS) is a chronic inflammatory disease with a significant contributor to mortality worldwide. Regulatory T cells (Tregs) are atheroprotective. However, the potential pathways and genes associated with atherosclerotic plaque progression in Tregs remain largely unknown. Therefore, this study aimed to identify critical target genes and pathways of Tregs associated with the progression of AS.

**Methods:**

The gene expression data and single cell RNA-seq data of AS were downloaded from the Gene Expression Omnibus (GEO) database. Initially, we quantified CD4^+^ T cell proportions in non-plaque and plaque tissues using cell infiltration by estimation of RNA sequences (CIBERSORT) analysis, identifying pivotal transcription factors regulating the number of Tregs in atherosclerotic plaque. Subsequently, we identified significantly differential expressed genes of Tregs during the progression of atherosclerotic plaque and investigated the key pathways and transcription factors for these differentially expressed genes using gene ontology (GO) analysis and transcription factor enrichment analysis (TFEA), respectively. We also employed high dimensional weighted gene co-expression network analysis (hdWGCNA) and cell-cell communication analysis to elucidate the modules and cascade reaction of Tregs in the progression of AS. The key genes diagnostic potential was assessed via receiver operating characteristic (ROC) curve analysis. Finally, the target genes were validated in AS model using Ldlr^−/−^ mice.

**Results:**

We found that the proportion of Tregs significantly decreased, and Th2 cells showed a significant increase in atherosclerotic plaque compared to that in non-plaque arterial tissues. The five transcription factors (TEFC, IRF8, ZNF267, KLF2, and JUNB), identified as key targets associated with the function and the number of Tregs driving the progression of AS, primarily regulate immune response, ubiquitination, cytokine production, and T-cell differentiation pathways. ZNF267 may mainly involve in regulating ubiquitination, TGF-beta, and MAPK pathways of Tregs to regulate the function and the number of Tregs during the progress of AS. Interestingly, we found that IRF8 and ZNF267 as potential biomarkers were upregulated in circulating CD4+ T cells in patients with atherosclerotic coronary artery disease. Moreover, we also found that the changes of the function and the number of Tregs could modulate endothelial cell and smooth muscle cell functions to counteract AS through ligand–receptor pairs such as the MIF signaling pathway. Finally, we validated that two of the five transcription factors were also upregulated in mice atherosclerotic plaque through AS model using Ldlr^−/−^ mice.

**Conclusion:**

Our results indicate that the transcription factors TEFC, IRF8, ZNF267, KLF2, and JUNB in Tregs could be potential targets for the clinical management of AS.

## Introduction

Cardiovascular disease is the leading cause of morbidity and mortality over the world, with an escalating incidence and mortality rate (1). Atherosclerosis (AS), a chronic inflammatory disease primarily affecting large and medium arteries, is the major contributor to cardiovascular diseases (2). Therefore, it is critical to investigate the pathogenesis of AS. The development of AS is a complex process involving the interaction of various cells, mainly endothelial cells (ECs), smooth muscle cells (SMCs), macrophages, and T cells. Among them, regulatory T cells (Tregs) have been reported to regulate ECs, SMCs, macrophages, and effector T cells to mediate the progression of AS (3).

Tregs are defined by their high expression of the CD25 and Foxp3 and can be differentiated from naïve CD4^+^ T cells in the periphery (4, 5). Multiple studies have shown that the number of Tregs is closely associated with the progression of AS (6). For example, a study using a vulnerable carotid plaque model ApoE deficiency (ApoE^−/−^) model mice demonstrated that increasing Treg numbers significantly reduced the relative content of macrophages and lipids while substantially increasing SMC and collagen levels within plaques. This changes led to a more stable plaque phenotype and reduced the incidence of plaque disruption (7). Tregs have also been found to favor M2 macrophage polarization to inhibit the inflammation phenotype and foam cell formation (8). Furthermore, Tregs suppress EC activation and the expression of adhesion molecules (E- and P-selectin) during atherogenesis (9). Clinically, low levels of circulating Tregs in humans are associated with a higher risk of acute coronary syndrome, whereas decreased lesional Tregs are linked to increased plaque vulnerability (10). Overall, the reduction of Tregs plays an important role in the progression of AS, and their decline in AS is thought to contribute to the chronic inflammation in the arterial wall that drives plaque progression (11–13).

However, the potential pathways and genes associated with atherosclerotic plaque progression in Tregs remain largely unknown. Hence, elucidating the intricate molecular mechanisms governed by Tregs in the context of AS holds the potential to substantively enhance the applicability of these biomarkers. Although Tregs are athero-protective, there is currently a lack of novel and robust pathways and biomarkers for Tregs associated with the progression of AS. This study used multiple bioinformatics analysis approaches to identify key biomarkers and further explore the underlying mechanism attributing to the altered biomarkers.

In this study, microarray datasets and single-cell sequencing data related to AS development were screened and downloaded from the Gene Expression Omnibus (GEO) database. First, we identified CD4^+^ T-cell populations in patient-matched proximal adjacent (PA) and entire calcified atherosclerotic core (AC) tissues based on the single-cell RNA sequencing (scRNA-seq) data (GSE159677). From this, we constructed the gene expression signal matrix of CD4^+^ T-cell subpopulations in atherosclerotic plaque to further calculate the differences in CD4^+^ T-cell subpopulations within bulk RNA-seq data of non-plaque and plaque tissues through cell infiltration by estimation of RNA sequences (CIBERSORT) analysis. Next, we performed gene co-expression network and correlation analysis to identify key targets affecting the number of Tregs during atherosclerotic plaque progression based on bulk RNA-seq data (GSE43292). Subsequently, we identified key pathways and transcription factors for the changes of function of Tregs between PA and AC tissues through differential expressed genes and transcription factor enrichment analysis. Additionally, we also constructed high-dimensional weighted gene co-expression network analysis (hdWGCNA) of Tregs in PA and AC tissues to identify the modules and genes driving the progression of AS. By integrating three bioinformatics approaches, we pinpointed potential targets for Tregs in AS progression and assessed their diagnostic potential using receiver operating characteristic (ROC) curve analysis. Furthermore, we also conducted cell-cell communication analysis, revealing the interaction pathways between Tregs and ECs, as well as between Tregs and SMCs. Finally, we validated these key targets in an AS model using Ldlr^−/−^ mice.

## Materials and methods

### Data collection

Microarray datasets and single-cell sequencing data were screened and acquired from the GEO database. The GSE43292 was obtained from the GEO database, which contained 32 normal carotid artery samples and 32 corresponding atherosclerotic plaque sample (14). We also downloaded the dataset GSE28829, which includes gene expression data of early-stage (n = 13) and advanced-stage (n = 16) atherosclerotic plaque (15). The GSE159677 was used for scRNA-seq analysis, which contained the single-cell transcriptome of AC (n = 3) plaques and PA (n = 3) portions of carotid artery tissue from patients undergoing carotid endarterectomy (16). The GSE9820 was used to investigate the expression of key genes in circulating CD4^+^ T cells between controls and patients with atherosclerotic coronary artery disease (CAD). The dataset of GSE9820 contained 14 controls and 20 patients with atherosclerotic CAD (17).

### Processing of scRNA-seq data

The RNA-seq data were downloaded from GSE159677. To obtain high-quality data for subsequent analysis, we performed a rigorous filtering procedure: cells that contained more than 500 expressed genes, less than 4,000 expressed genes, and Percentage of Cells with Mitochondrial Transcripts (pctMT) < 15% passed the cell quality filtering. The scRNA-seq data were created by the Seurat object to perform cell normalization and scale using the Seurat package in R software. The top 2,000 variable features of each sample were analyzed after normalization. To construct Principal Component Analysis (PCA), we selected the top 20 principles for t-distributed stochastic neighbor embedding and uniform manifold approximation and projection dimensional reduction. The graph-based cluster method acquired the unsupervised cell clusters based on the top 20 PCA principles (resolution = 0.5). The function of “FindALLMarkers” was used to identify marker genes of each cluster using R software. The marker genes of each cluster were defined as those with the Wilcoxon rank sum test algorithm, logfc.threshold > 0.25, and adjusted p-value < 0.05.

### Evaluation of immune cells in atherosclerosis plaque

To identify the different immune cell compositions in AS plaque, we performed the analysis with the help of the CIBERSORT algorithm and LM22 leukocyte signature matrix as the input matrix of reference gene expression signatures (18). Moreover, we also constructed a personalized signature matrix as the input matrix of reference gene expression signatures to calculate the different immune cell compositions. The bulk-RNA sequence data of non-plaque and plaque tissues from GSE43292 was obtained for CIBERSORT analysis. The abundance and proportion of immune cell members among different groups were fully analyzed and visualized using R software.

### Weighted gene co-expression network analysis for microarray data and hdWGCNA analysis for single-cell datasets

WGCNA for microarray data were constructed and analyzed using the WGCNA package in R (19). This study used 14,256 genes to construct the gene co-expression network in non-plaque and plaque tissues. A power threshold of 8 was selected to calculate the weighted adjacency matrix, with the thresholding parameter defined using a scale-free topology with a cutoff R^2^ = 0.8. This cutoff was chosen to be the closest to or slightly above 0.8 to establish a biologically meaningful and stable network for further analysis. Next, hierarchical clustering analysis was performed to calculate topological overlap measures (TOMs) among genes and assign the genes to different modules. We identified gene modules using the “hybrid” method with parameters mergeCutHeight = 0.25 and minModuleSize = 30. Subsequently, we used TOMs to reveal the interconnectedness and functional relationships among genes within the network (20). WGCNA for datasets of GSE43292 was published in a previous study (21). According to the characteristics of scRNA-seq data, we used the hdWGCNA method to construct a gene co-expression network (22). To reduce the sparsity and noise inherent in single-cell data for gene-gene correlation, we used the hdWGCNA method to collapse highly similar cells into “beta cells” to reduce sparsity while retaining cellular heterogeneity and by allowing for a modular design to perform separate network analyses in specified cell populations following the parameters of WGCNA. The key genes in each module are the following stringent criteria: genes that exhibit an absolute correlation coefficient >0.90 with the eigengene of the enriched module and genes that are significantly correlated with the number of Tregs and the stage of atherosclerotic plaque progression.

### Gene set enrichment and differential gene expression analysis

Gene set enrichment analysis (GSEA) was performed at a single-cell resolution based on c5.bp.v7.0.symbols.gmt [Gene ontology biological process (GOBP)] and c2.cp.kegg.v7.0 symbols.gmt [Kyoto Encyclopedia of Genes and Genomes (KEGG)] reference gene sets using the GSEA package in R (23, 24). Briefly, GSEA is a computational method determining whether a priori-defined set of genes shows statistically significant, concordant differences between two biological states. Moreover, to identify differentially expressed genes (DEGs) for Tregs in atherosclerotic plaque at PA and AC stages from scRNA-seq data, we used the function “FindMarkers” to identify DEGs using R software. DEGs were defined as those with Wilcoxon rank sum test algorithm; logfc.threshold > 0.25 and p-value < 0.05.

### GO enrichment analyses

GO pathway enrichment analysis was performed using “clusterProfile” package in R software (25). The Benjamin–Hochberg approach was used to correct multiple tests and select the significant terms and pathways. The adjusted p-value < 0.05 was used as a significance threshold for the enriched terms and pathways for DEGs or marker genes of cell types.

### Transcription factor enrichment analysis (TFEA) by orthogonal omics integration

To obtain the key transcription factors of DEGs, we performed TFEA by orthogonal omics integration (ChEA3) (26). TFEA prioritizes transcription factors based on the overlap between given lists of DEGs and previously annotated transcription factor targets assembled from multiple resources. The multiple resources include Chromatin Immunoprecipitation Sequencing (ChIP-seq) experiments from ENCODE, ReMap, and individual publications; co-expression of transcription factors with other genes based on processed RNA-seq from GTEx and ARCHS4; co-occurrence of transcription factors with other genes by examining thousands of gene lists submitted to the tool Enrichr; and gene signatures resulting from single transcription factor perturbations followed by genome-wide gene expression experiments.

### Logistic regression models with the ROC curve

The logistic regression model was constructed using glm in R. The key genes were identified as predictive variables, and the sample type with early-stage or advanced stage was considered as a binary responsive variable based on the datasets of GSE43292 and GSE28829. The three-fold cross-validation was performed to validate the accuracy of the logistic regression models by caret package in R. The ROC curves were generated to evaluate the sensitivity and specificity of the logistic regression models. The average area under the curve (AUC) was calculated to assess the models’ accuracy.

### Cell-cell communication analysis

We used CellChat (1.1.0), a public repository of ligands, receptors, cofactors, and their interactions for the inference and analysis of cell-cell communication (27). The versatile and easy-to-use toolkit CellChat and a web-based Explorer help discover novel intercellular communications and build cell-cell communication atlases. The expression values were averaged within each single-cell cluster population. The p-value < 0.05 is the significance threshold of cell communication based on the molecule interactions.

### Aortic tissue collection of animal model

The 6 to 8-week-old male ApoE^−/−^ mice were randomly divided into high-fat diet group (n = 6) and normal chow group (n = 6). All mice were fed for 16 weeks and then sacrificed after fasting for 8 h. The aortic tissues were removed from the ascending aorta to the ileal bifurcation and snap-frozen in liquid nitrogen for RNA analysis.

### RNA extraction and quantitative real-time PCR

Total RNA from aortic tissues was extracted using TRIzol reagents. cDNA was synthesized using the PrimeScriptTM RT Master Mix kit (Takara) following the manufacturer’s instructions. Quantitative real-time PCR was performed using the SYBR^®^ Premix Ex TaqTMII (Takara) after setting up the appropriate protocol. The *β*-*action* was used as the internal control. Relative gene expression was calculated using the ΔΔC_t_ method.

### Statistical analysis

Data statistical analysis and visualization were performed using R software. The correlation among different continuous variables was obtained by Spearman’s correlation coefficient. Multiple comparisons between categorical variables using ANOVA analysis and t-test were applied for statistical analysis between two clusters or cell types. A p-value <0.05 was considered statistically significant.

## Results

### Dynamic changes of T-cell subpopulations during the progression of atherosclerosis plaque

**Figure 1:**
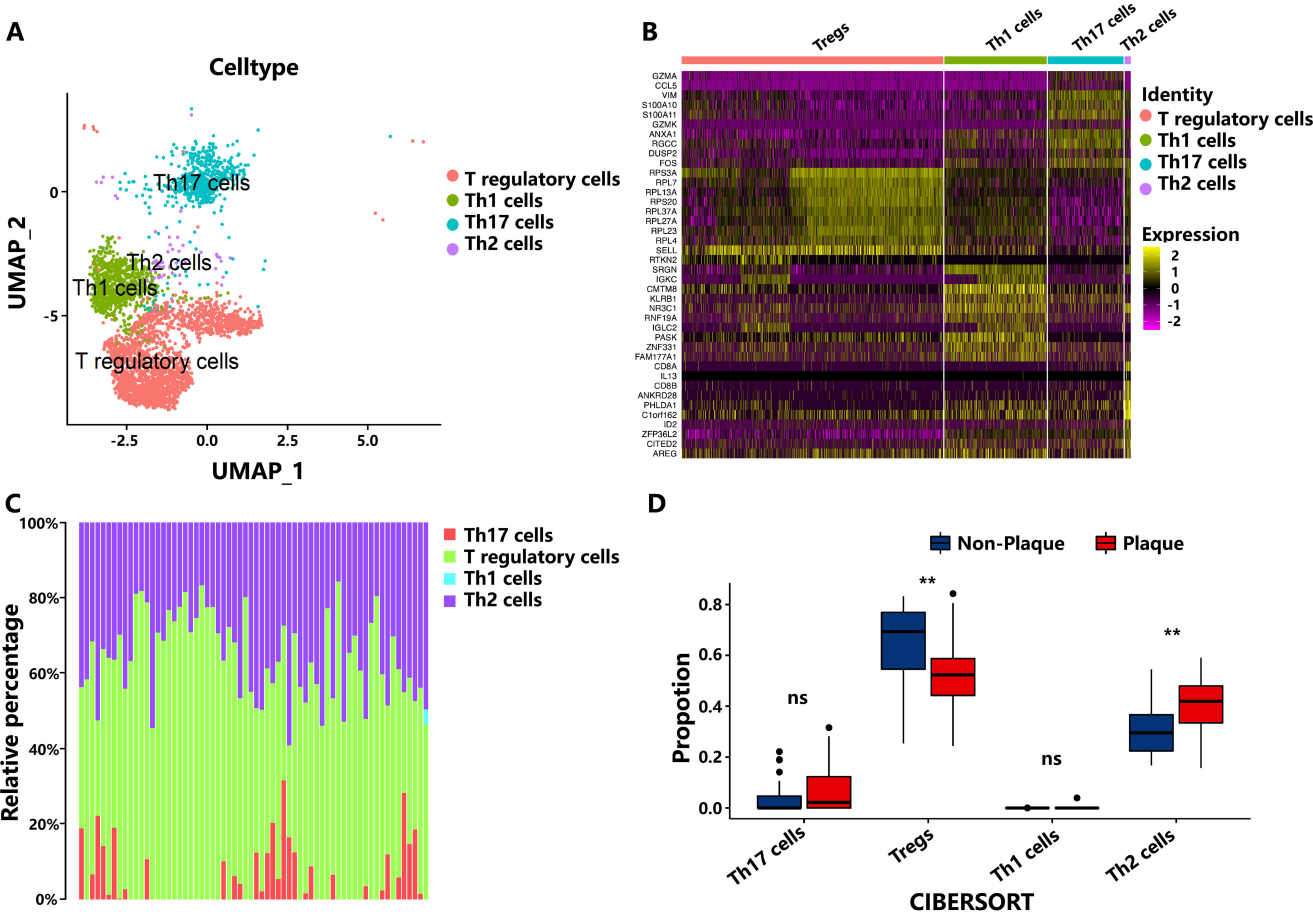
The number of subpopulations of T cells during the progression of atherosclerotic plaque. **(A)** Cell-type assignment following uniform manifold approximation and projection (UMAP)–based visualization of expression differences for CD4^+^ T cells from the single-cell transcriptome of entire calcified atherosclerotic core and patient-matched proximal adjacent portions of carotid artery tissue. **(B)** Top 10 markers of cell clusters. **(C)** The proportion of cell types from the non-plaque to plaque stage. **(D)** Boxplot of the proportion of cell types from the non-plaque to plaque stage. ** indicates P value less than 0.01 (**P < 0.01). “ns” means there is no significant difference between the two groups.

We identified that the subpopulations of CD4^+^ T cells, including Th1, Th2, Th17, and Tregs within PA and AC tissue atherosclerotic plaques based on the scRNA-seq dataset of GSE159677 (**Figures 1A, B**). Then, the gene expression signal matrix of CD4^+^ T-cell subpopulations (GSE159677) was constructed to further calculate the number of CD4^+^ T-cell subpopulations in non-plaque and plaque tissues based on the dataset of GSE43292 through CIBERSORT analysis (**Supplementary Table S1**). Tregs exhibited significant reduction during the progression of atherosclerotic plaque after the method of deconvolution integral to identify the changes in CD4^+^ T-cell subpopulation between non-plaque and plaque tissues of bulk RNA-seq data, in which the result is consistent with the analysis of LM22 leukocyte signature matrix (**Figures 1C, D**; **Supplementary Figure S1**). Moreover, we also found that Th2 cells showed a significant increase in atherosclerotic plaque (**Figures 1C, D**). However, Th1 and Th17 cells did not show significant changes in PA and AC stages. These results indicated that the proportion of Th2 and Tregs have significantly changed during the progression in AS.

### The key pathways and transcription factors affecting the number of Tregs during the progression of atherosclerotic plaque

**Figure 2:**
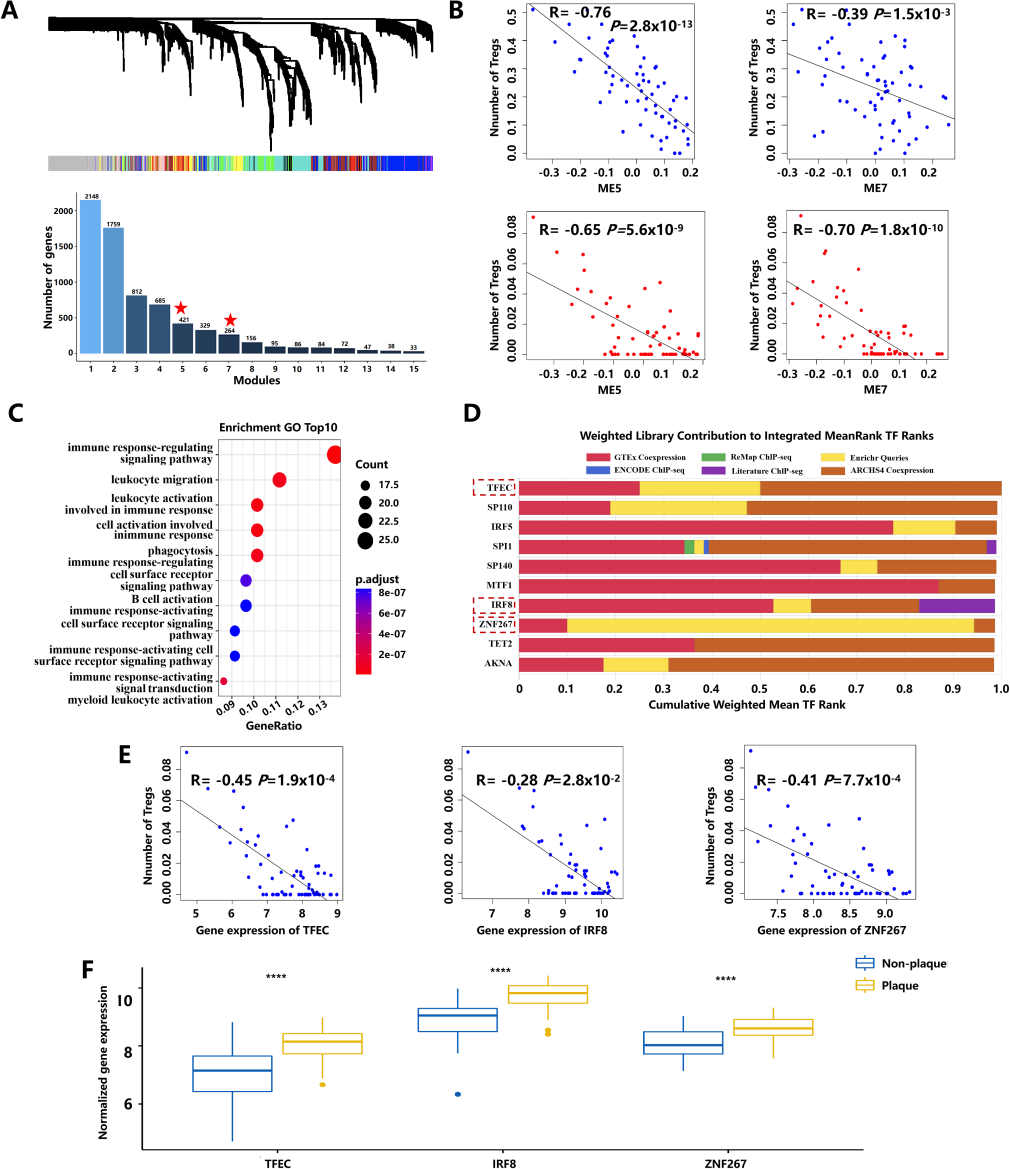
The key pathways and transcription factors affect the number of Tregs during atherosclerotic plaque progression. **(A)** The gene dendrogram and the bar plot of gene size in each module were based on weighted gene co-expression network analysis. The asterisk represents the module that displayed modular differential connectivity from non-plaque to plaque. **(B)** Scatter plot showing the correlation between modules and the number of Tregs. Blue represents the number of Tregs calculated based on the LM22 leukocyte matrix. Red represents the number of Tregs calculated based on a personalized signature matrix. **(C)** The bubble diagram of GO-BP enrichment analyses of the key genes. Dot sizes represent counts of enriched genes, and dot colors represent adjusted p-values. **(D)** The top 10 transcription factors of 209 key genes during atherosclerotic plaque progression. **(E)** Scatter plot showing the correlation between transcription factors and the number of Tregs. **(F)** Boxplots of the expression of three transcription factors with extremely changed from non-plaque to plaque tissue. **** indicates P value less than 0.0001 (****P < 0.0001).

To investigate key genes influencing the number of Tregs during atherogenesis, a weighted gene co-expression network (WGCNA) was constructed (**Figure 2A**) using the dataset of GSE43292. The correlation analysis between Treg numbers and modules based on LM22 leukocyte signature matrix and personalized signature matrix revealed that modules 5 and 7 were significantly negatively correlated with the number of Tregs (**Figure 2B**). Within module 5, 209 key genes significantly associated with Treg numbers, and atherosclerotic plaque stages were identified using stringent screening criteria. Gene Ontology (GO) enrichment analysis showed that these key genes were mainly enriched in the immune system, leukocyte activation, and migration (**Figure 2C**; **Supplementary Table S2**). Ten transcription factors with the highest scores were selected in module 5 using Transcription factor enrichment analysis (TFEA) by orthogonal omics integration (**Figure 2D**; **Supplementary Table S3**). Among them, transcription factor EC (TFEC), interferon regulatory factor 8 (IRF8), and zinc finger protein 267 (ZNF267) were also found within the 209 key genes, showing significant associations with Treg numbers and plaque progression (**Figures 2E, F**). These findings suggest that TFEC, IRF8, and ZNF267 may serve as critical transcription factors regulating Treg numbers during atherosclerotic plaque progression.

### The key pathways and transcription factors influencing the change of Tregs function in the progression of atherosclerotic plaque

**Figure 3:**
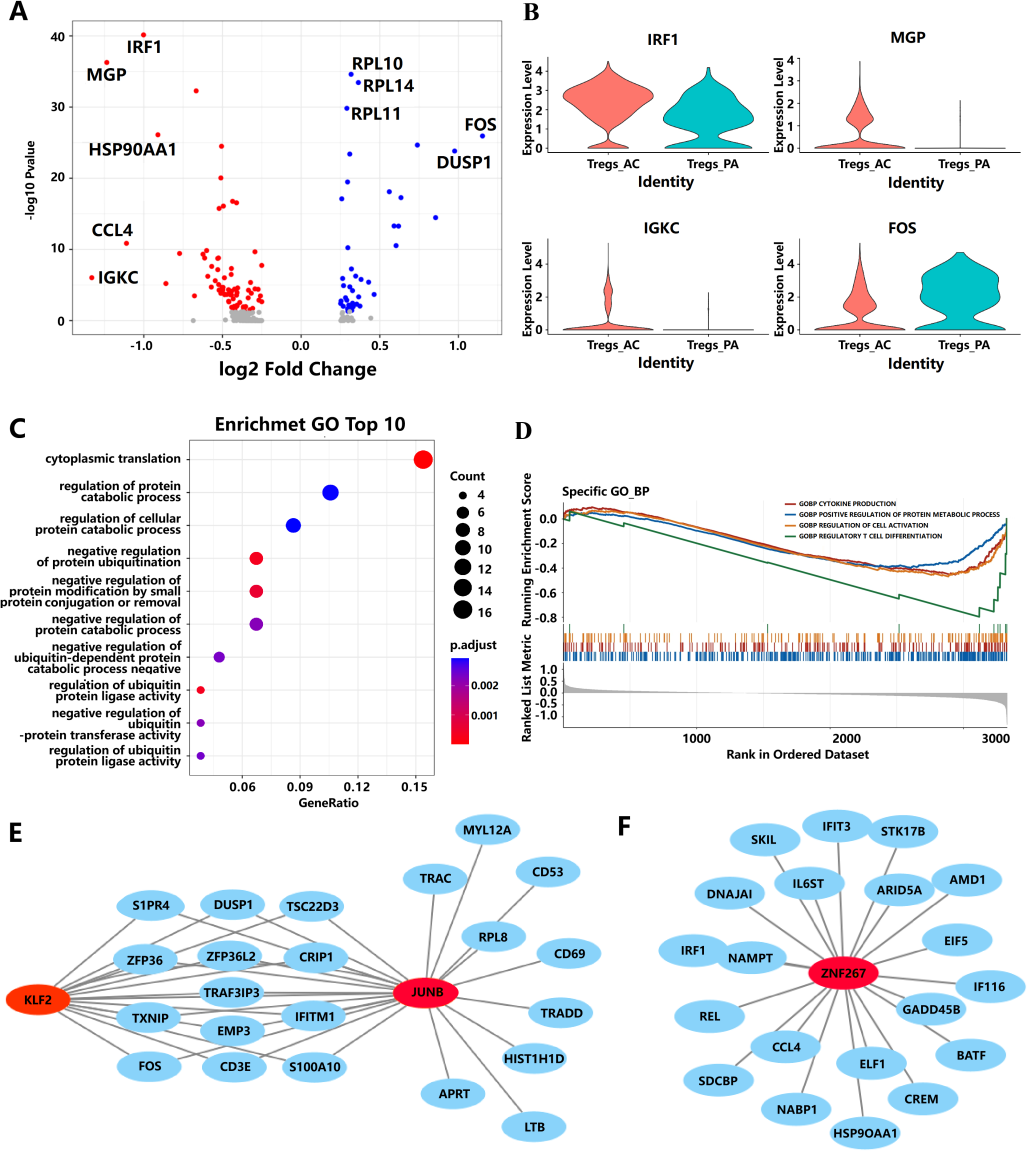
The potential pathways and biomarkers for Tregs during the progression of AS. **(A)** Representation of the DEGs comparing Tregs from the PA and AC stages. **(B)** Violin plots of the expression of four genes have changed significantly from the PA stage to the AC stage. **(C)** The bubble diagram of GO-BP enrichment analyses of the significantly differential expressed genes in Tregs from PA to AC stage. **(D)** Gene sets show significant enrichment between the PA and AC stages in Tregs. **(E, F)** Interaction network diagram of transcription factors and target genes. The gene marked by red represents the transcription factor. Blue represents target genes.

A total of 120 DEGs were found in Tregs of PA and AC tissues (**Figure 3A**). The top five upregulated genes [IRF1, Immunoglobulin kappa constant (IGKC), chemokine (C-C motif) ligand 4 (CCL4), Matrix Gla protein(MGP), Heat shock protein 90kDa alpha (cytosolic), class A member 1(HSP90AA1)] and downregulated genes [Fos proto-oncogene (FOS), Dual-specificity phosphatase 1 (DUSP1), Ribosomal protein L10 (RPL10), Ribosomal protein L14 (RPL14), Ribosomal protein L11 (RPL11)] in PA and AC stages, which displayed an extremely high fold change in differential expression, are displayed in **Figure 3A**. Of them, four extremely changed genes (IRF1, MGP, IGKC, and FOS) were shown in the violin diagram (**Figure 3B**). These 120 DEGs were mainly involved in cytoplasmic translation (*P* = 2.49 × 10^−13^), regulation of ubiquitin process (*P* = 2.8 × 10^−4^), and regulation of protein catabolic process (*P* = 2.0 × 10^−3^) following GO enrichment analysis. Interestingly, 50% of the top 10 enrichment pathways were involved in regulating protein ubiquitination (**Figure 3C**; **Supplementary Table S4**). GSEA analysis identified that the gene sets of cytokine production, regulation of protein metabolic process, cell activation, and regulation of T-cell differentiation were significantly enriched in AC tissue (**Figure 3D**; **Supplementary Table S5**).

To identify potential key transcription factors responsible for the differential gene expression profiles between PA and AC stages in AS, we performed TFEA by orthogonal omics integration of the 120 DEGs (43 upregulated genes and 77 downregulated genes, respectively) in the AC stage (**Supplementary Figure S2**). Transcription factors were ranked on the basis of the mean rank (average integrated ranks across libraries), and the top 10 transcription factors with the lowest scores were subjected to a common gene analysis with the 120 DEGs. Among them, three transcription factors (KLF2, JUNB, and ZNF267) were found to overlap with these DEGs (**Figures 3E, F**). The interaction network diagram of these three transcription factors and target genes (**Figures 3E, F**) suggests that KLF2, JUNB, and ZNF267 may play potential roles in driving the differential gene expression between the PA and AC stages during the progression of AS.

### The hdWGCNA analysis revealed key modules and genes of Tregs associated with atherosclerotic plaque

**Figure 4:**
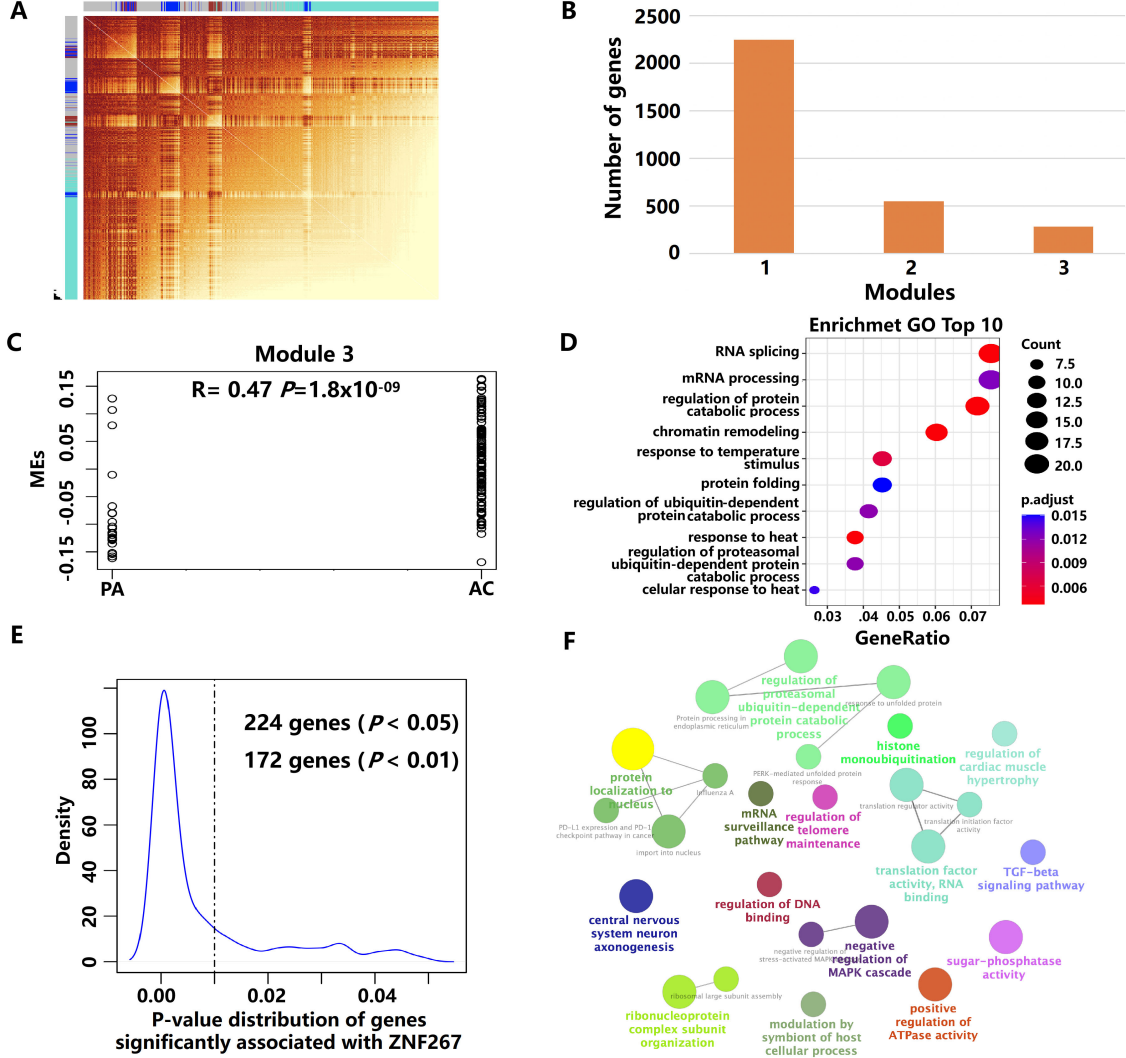
The key modules and genes of Tregs using hdWGCNA analysis. **(A)** The high-dimensional weighted gene co-expression network (hdWGCNA) analysis in Tregs. **(B)** The boxplot showed the number of genes in each module. **(C)** The results of GO enrichment analysis of genes in module 3. **(D)** Scatter plot showing the correlation between modules and the plaque stage in module 3. **(E)** The p-value distribution of genes was significantly associated with ZNF267. **(F)** The results of GO enrichment analysis of 172 genes.

The gene co-expression network for Tregs in different plaque stages was constructed using scRNA-seq data of PA and AC stages by hdWGCNA analysis (**Figure 4A**). Three gene co-expression modules in Tregs were identified (**Figure 4B**). Of them, module 3 was highly correlated with the stages of AS (**Figure 4C**). GO enrichment analysis revealed that the genes in module 3 were primarily involved in the regulation of protein catabolic processes and ubiquitin-dependent protein catabolic processes (**Figure 4D**). After rigorous filtering, ZNF267 and RYBP were identified as the key targets driving module 3 in the progression of atherosclerotic plaque based on hdWGCNA analysis (**Supplementary Table S6**). To elucidate the primary role of ZNF267 in plaque progression, we identified 172 genes, including RYBP gene, that were significantly correlated with ZNF267 (*P* < 0.01). These genes were predominantly associated with pathways regulating the ubiquitination, Transforming growth factor beta (TGF-beta) signaling pathway, and Mitogen-activated protein kinase (MAPK) cascade pathway (**Figures 4E, F**). These findings indicate that ZNF267 may mainly regulate module 3, influencing ubiquitination, TGF-beta, and MAPK pathways, thereby mediating the progression of AS.

### The key transcription factors as potential therapeutic targets in AS

**Figure 5:**
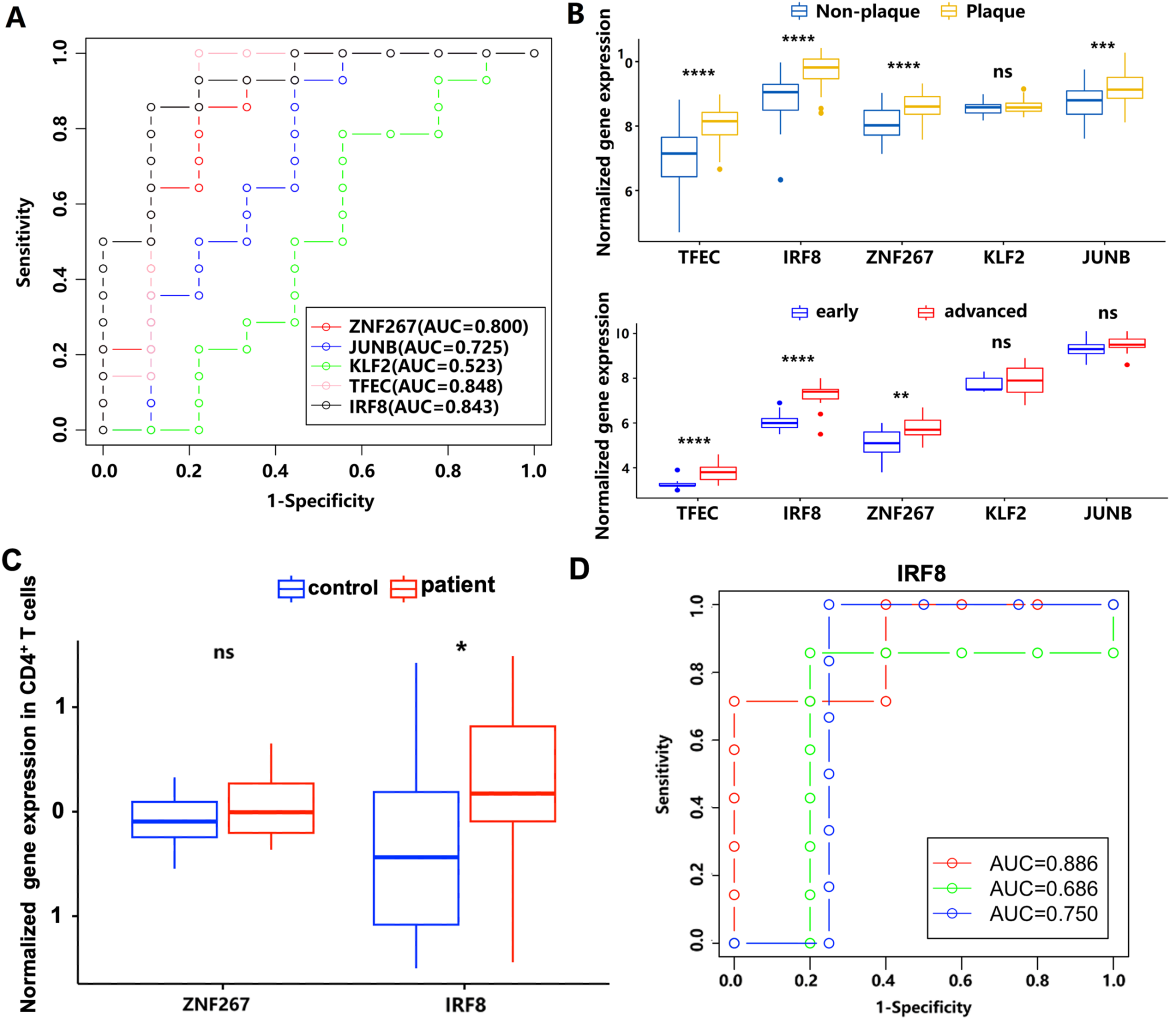
The diagnostic power of ZNF267, JUNB, KLF2, TFEC, and IRF8 in AS by ROC curve. **(A)** The receiver operating characteristic curve analysis of ZNF267, JUNB, KLF2, TFEC, and IRF8. The different colors represent the respective genes. **(B)** The expression differences of the five target genes between the non-plaque and plaque stages or the early and advanced stages in AS. **(C)** The expression level of ZNF267 and IRF8 in circulating CD4^+^ T cells is compared between controls and patients with atherosclerotic CAD. **(D)** The receiver operating characteristic curve of IRF8. The different colors represent the results from three cross-validation processes. * indicates P value less than 0.05 (*P < 0.05); ** indicates P value less than 0.01 (**P < 0.01); *** indicates P value less than 0.001 (***P < 0.001); **** indicates P value less than 0.0001 (****P < 0.0001).

To obtain the potential predictive value of key gene markers in AS, we generated ROC curves (**Figure 5A**). The AUCs for the genes ZNF267, JUNB, KLF2, TFEC, and IRF8 were 0.800, 0.725, 0.523, 0.848, and 0.843, respectively (**Figure 5A**). Among these, ZNF267, TFEC, and IRF8 (AUC ≥ 0.8) were upregulated when comparing non-plaque and plaque tissues, as well as early and advanced stages, in the GSE43292 and GSE28829 datasets (**Figure 5B**). Interestingly, we found that IRF8 and ZNF267 were also upregulated in circulating CD4^+^ T cells of patients with atherosclerotic CAD (**Figure 5C**). Using a logistic model validated by three-fold cross-validation, the AUC values for IRF8 in three independent validation sets were 0.886, 0.686, and 0.750, with an average AUC of 0.774 (**Figure 5D**). These results suggest that IRF8 could be used as a potential biomarker of AS in the circulating CD4^+^ T cells and atherosclerotic plaque.

### Cell-cell communication analysis revealed cascade reaction of Tregs to ECs and SMCs in the progression of atherosclerotic plaque

**Figure 6:**
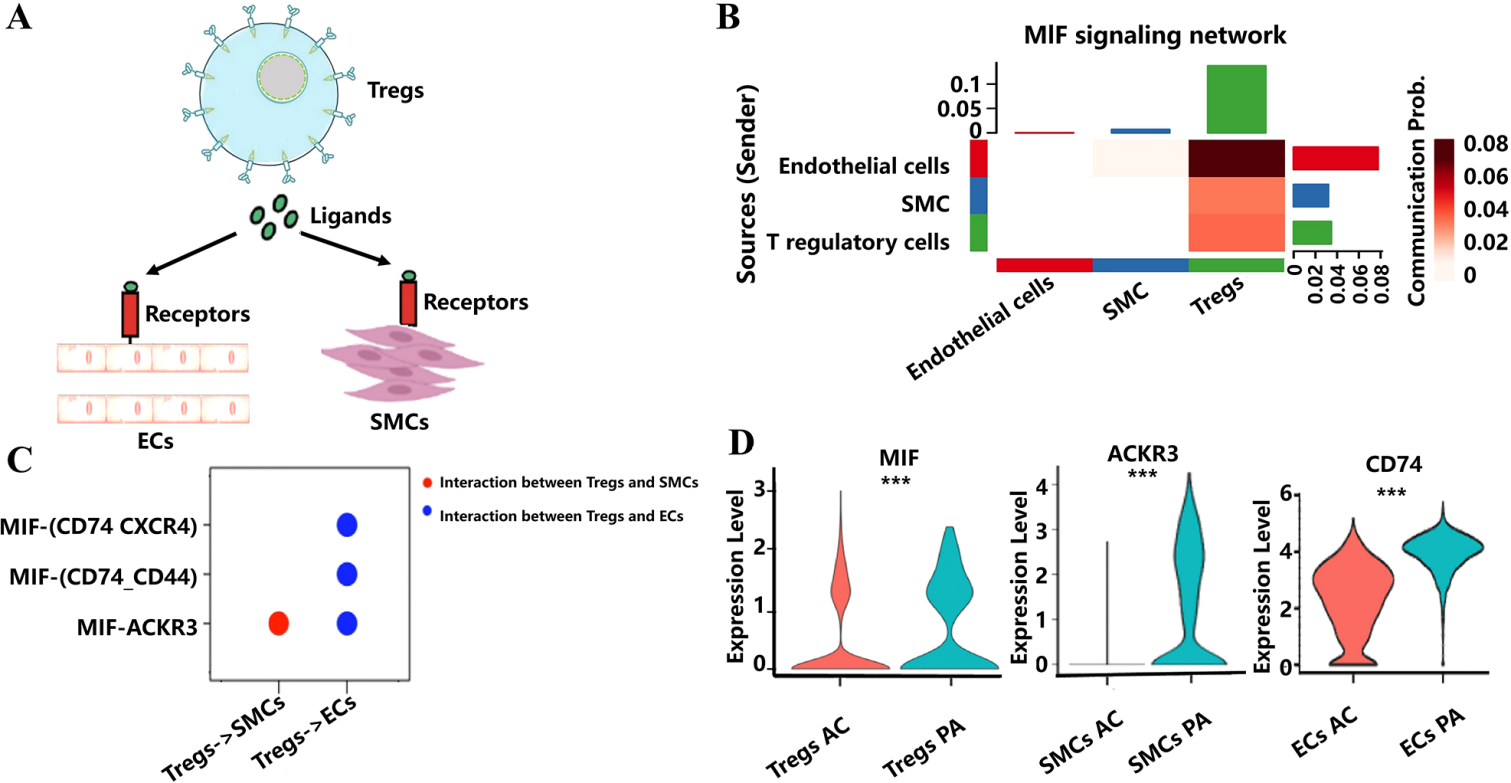
Cell-cell communication analysis revealed cascade reaction of Tregs to ECs and SMCs. **(A)** Schematic diagram of Tregs modulate the function of ECs and SMCs through ligand–receptor pairs. **(B)** The heat map shows the involvement of signaling pathways in the interaction between Tregs, ECs, and SMCs. **(C)** Displaying ligand–receptor pairs for communication between Tregs and ECs, as well as between Tregs and SMCs. **(D)** Violin plots of the expression of ligand–receptor pairs have changed significantly from the PA to the AC tissue. *** indicates P value less than 0.001 (***P < 0.001).

To obtain insights into how Tregs regulate the function of macrophages, ECs, and SMCs in combating AS through ligand–receptor pairs, this study performed cell-cell communication analysis to uncover the possible cascade reaction of Tregs to macrophages, ECs, and SMCs during the atherosclerotic plaque progression. We retained ECs and SMCs for cell-cell communication analysis with Tregs, using filtering criteria with a cell count greater than 10 in each group (**Figure 6A**). Through cell-cell communication analysis, we found that Migration Inhibitory Factor (MIF) signaling network including ligand–receptor pairs like MIF-ACKR3, MIF-(CD74_CD33), and MIF-(CD74_CXCR4) could be a potential bridges to modulate the function of ECs and SMCs in combating AS (**Figures 6B, C**). Among these ligand–receptor pairs, MIF, ACKR3, and CD74 were found to be significantly decreased in Tregs, SMCs, and ECs, respectively, during AS progression. This results indicated that changes in the function and the number of Tregs could influence EC and SMC functions through these ligand–receptor interactions, potentially counteracting the effects of AS (**Figure 6D**).

### Validation of key transcription factors expression in atherosclerotic plaque

**Figure 7:**
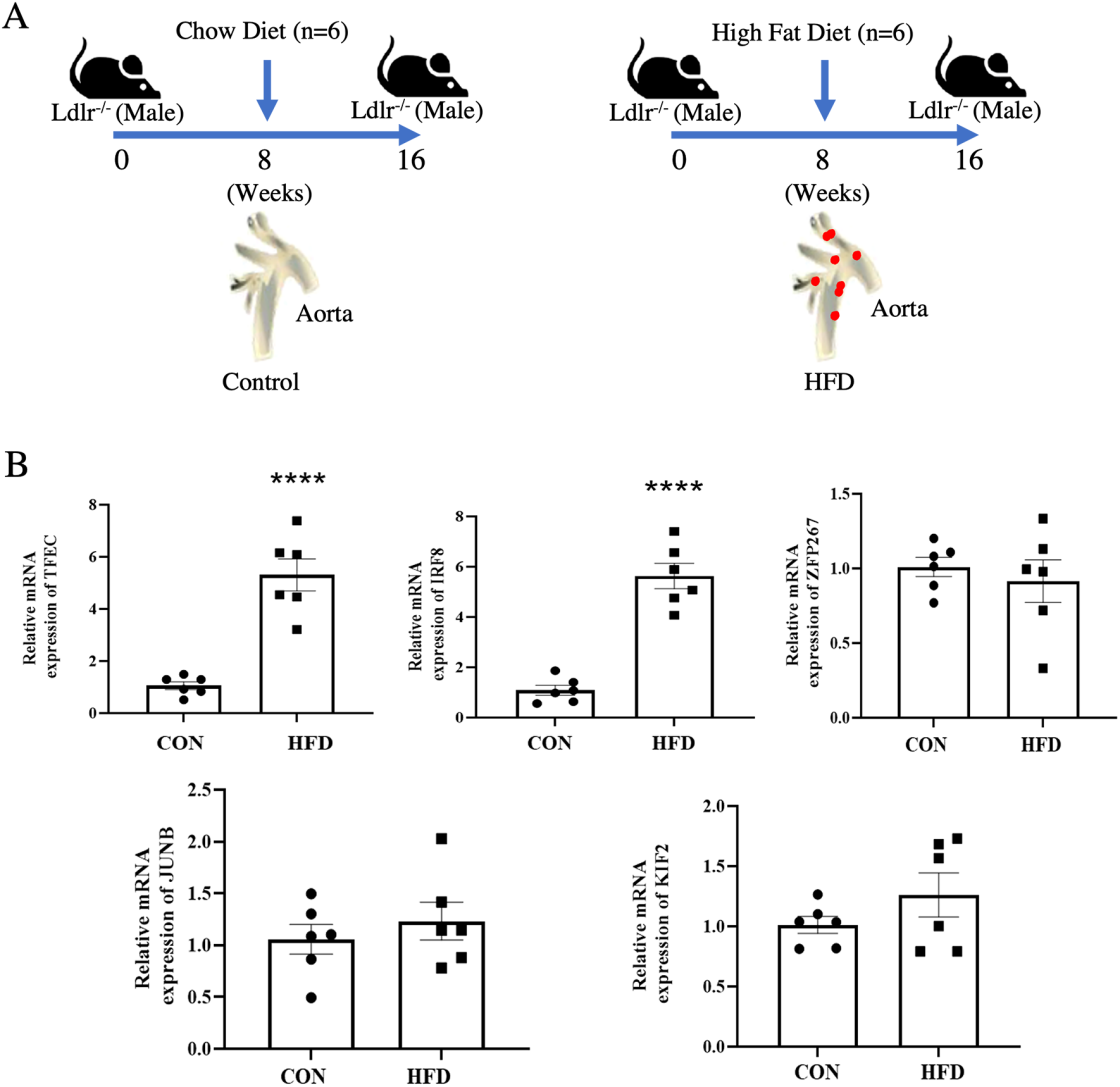
Validation of key transcription factors expression in atherosclerotic plaques. **(A)** Schematic diagram of the AS model in Ldlr^−/−^ mice. **(B)** The expression level of five key transcription factors in Ldlr^−/−^ mice aortic tissues. **** indicates P value less than 0.0001 (****P < 0.0001).

To validate the expression of the five transcription factors in atherosclerotic plaque, we constructed a mouse model by Ldlr^−/−^ mice with high fat-diet for 16 weeks (**Figure 7A**). We found that IRF8 and TFEC were significantly upregulated in atherosclerotic plaque group compared to that in the control group (*P* < 0.05) using quantitative real-time PCR method. Although JUNB and KLF2 were also upregulated in the atherosclerotic plaque group, the changes were not statistically significant (**Figure 7B**).

## Discussion

AS is a complex chronic inflammatory disease, and both the adaptive and innate responses of the immune system are activated in AS (28). Tregs play an important role in AS (3), which are athero-protective and could function as target cells in AS (29). However, the relevant mechanisms of key genes and pathways in Tregs remain largely unknown. This study investigated the composition of CD4^+^ T-cell subpopulations and found that Tregs and Th2 significantly changed during the progression of atherosclerotic plaque. Moreover, we identified potential key pathways and targets that affect the number and function of Tregs during AS progression. We also identified the ligand–receptor pairs such as the MIF signaling pathway as potential bridges of Tregs to modulate EC and SMC functions to counteract AS. Finally, we validated the key targets using AS in mice. This study provides valuable for understanding gene regulation underlying the mechanism of Tregs in humans and provides biomarkers for predictive diagnosis, targeted prevention, and personalized treatment of the disease.

In this study, the proportion of Th2 cells significantly increased, whereas Th1 cells showed no significant changes during the progression of AS plaques, indicating an imbalance in the Th1/Th2 ratio. Several studies have shown that Th1 cells sustain inflammation via the secretion of interferon-γ and stimulate monocyte/T-cell recruitment into plaques, increase macrophage foam cell formation, and activate dendritic cells (30–32). The role of Th2 cells in the development of AS is still controversial, depending on the stage or location of the lesion, the type of Th2-secreting factor, and the experimental model (33). Previous studies have reported that the immune balance of Th1/Th2 involves the development of AS (34). Our results emphasize the importance of the balance between Th1 and Th2 in the progression of AS. Tregs are athero-protective (35), whereas the roles of Th17 cells in AS are controversial. Both pro-atherosclerotic and anti-atherosclerotic functions of Th17 cells have been reported (36). Th17 cells and their secreted cytokines have pro-inflammatory effects, whereas Treg cells and their secreted cytokines have anti-inflammatory effects (37). The disruption of Treg/Th17 cell balance is associated with disease progression (38, 39). In this study, the number of Tregs and the Tregs/Th17 significantly decreased, and Th17 cells showed no significant changes during the progression of AS, indicating that the immune balance of Tregs/Th17 was destroyed and the function of Tregs was inhibited during the progression of AS, and the changes in the balance of these cell types may serve as potential target cells for preventing and treating atherosclerotic plaque progression.

The number of Tregs has been reported involved in the progression of AS (3). Few studies reported key genes and pathways regulating the number of Tregs in AS. Immune cell infiltration and immune-related pathways participating in the progression of carotid atherosclerotic plaques were reported on the basis of the GSE43292 and GSE28829 datasets using the SM22 signature matrix through CIBERSORT analysis (40). Compared to this previous study, our study calculated the proportion of Tregs based on the SM22 signature matrix and personalized signature matrix through CIBERSORT analysis. The personalized signature matrix of the subpopulations of CD4^+^ T cells matrix was constructed on the basis of scRNA-seq of atherosclerotic plaque that was first constructed and applied in the analysis of CD4^+^ T-cell immune infiltration in bulk RNA data. We estimated the proportion of Tregs in the early and advanced stages of atherosclerotic plaque based on two signature matrixes and identified three transcription factors (IRF8, TFEC, and ZNF267), which were significantly associated with the number of Tregs and plaque progression that may affect the proportion of Tregs in atherosclerotic plaque. IRF8-mediated differences in macrophage foam cell formation, cholesterol ester content, and macrophage migration are involved in the progression of AS (41). In recent study, IRF8 was reported as a critical biomarker for immune infiltration in AS advance (42). TFEC is a member of the microphthalmia-associated transcription factor family (43), which may promote the development of coronary artery disease (44). ZNF267 is a member of the Kruppel-like transcription factor family that regulates various biological processes such as cell proliferation and differentiation (45). TFEC and ZNF267 have not been reported associated with the number of Tregs in atherosclerotic plaque. In this study, we further confirmed that IRF8 as a critical biomarker in AS and first reported that TFEC and ZNF267 may regulate the number of Tregs and participate in the regulation of atherosclerotic plaque. Of them, IRF8 and ZNF267 were upregulated in circulating CD4^+^ T cells of patients with atherosclerotic CAD. Notably, IRF8 and ZNF267 could be a biomarker in circulating CD4^+^ T-cell for predictive diagnosis, targeted prevention, and personalized treatment of the atherosclerotic CAD. The new biomarkers could provide additional molecular-level insights that complement existing tools, such as capturing inflammatory and immune-related changes of Tregs in atherosclerotic progression. In addition, these biomarkers may enhance diagnostic accuracy when integrated into panels with established markers like C-reactive protein and facilitate inclusion in blood tests to monitor AS in a non-invasive manner. Finally, stratifying patients based on these biomarkers might help identify those who would benefit from specific anti-inflammatory or Treg-modulating therapies.

After analyzing the single-cell transcriptome data of Tregs isolated from the PA and AC, we identified 120 DEGs. Among them, the top five upregulated genes (IRF1, IGKC, CCL4, MGP, and HSP90AA1) are mainly involved in the immune system pathway, whereas the top five downregulated genes (FOS, DUSP1, RPL10, RPL14, and RPL11) are mainly involved in the T-cell differentiation pathway (43–45). The gene sets of cytokine production, regulation of protein metabolic process, cell activation, and regulation T-cell differentiation were significantly enriched in the AC stage following scRNA-seq data through GSEA analysis. These results indicated that Tregs may regulate the production of cytokine during the progression of atherosclerotic plaque and reduce the number of Tregs by regulating T-cell differentiation. The result of GO enrichment analysis revealed that the 120 DEGs are mainly enriched in the regulation of the ubiquitin process, cytoplasmic translation, and regulation of the protein catabolic process. Ubiquitination is a multistep post-translational protein modification that participates in many important cellular processes. Emerging evidence suggests that ubiquitination plays important roles in the pathogenesis of AS in many ways, including regulation of vascular inflammation, EC and vascular SMC function, lipid metabolism, and atherosclerotic plaque stability (46). In this study, we reported the ubiquitination regulation of Tregs related to atherosclerotic plaque. On the basis of these results, we assumed that ubiquitination mainly affects Tregs and then regulates other cells, including macrophages, ECs, and vascular SMCs, to contribute to the progression of atherosclerotic plaque.

Moreover, KLF2, JUNB, and ZNF267 transcription factors were identified as the critical targets that regulated the 120 DEGs. KLF2 is an important transcription factor involved in regulating the process of AS, including KLF2 that regulates endothelial pro-inflammatory activation (47), KLF2 that regulates SMC migration (48), and KLF2 that regulates regulatory T-cell production (49). JUNB also has been reported playing a crucial role in the development of Tregs by promoting IL-2 signaling (50). Interestingly, we identified ZNF267 as a key transcription factor regulating the co-expression network module with ubiquitination function significantly associated with AS plaque progression in Tregs. ZNF267 has been reported involved in tumor cell proliferation and migration in the previous study (51). However, ZNF267 regulating Tregs involved in atherosclerotic plaque has not been reported. Through annotation of gene functions of potential regulatory target genes, ZNF267 is mainly involved in the function of ubiquitination. Ubiquitination plays a crucial role in regulating the functions of Tregs by modulating their stability, proliferation, and suppressive capabilities (52, 53). Previous studies have shown that deubiquitinates Recombinant Ubiquitin Specific Peptidase 7 (USP7) and USP44 can regulate FOXP3 stability (54, 55). On the basis of these findings, we hypothesized that ZNF267 may inhibit the activity of deubiquitinases during AS progression, thereby reducing FOXP3 protein expression and diminishing both the anti-inflammatory activity and the numbers of Tregs.

Atherosclerotic plaque is a complex cellular microenvironment involving multiple cells. MIF signaling pathways were first reported as novel bridges, implying how Tregs modulate ECs and SMCs to fight against AS. Among the ligand–receptor pairs from the MIF signaling pathway, MIF has been reported to promote AS by mediating leukocyte recruitment, lesional inflammation, and suppressing athero-protective B cells (56). Endothelial ACKR3 facilitates the adhesion of immune cells to the vascular endothelium, contributing to the progression of AS (57). ACKR3 plays a pivotal role in regulating monocyte-macrophage function and inducing pro-inflammatory signaling pathways in macrophages (58). Previous studies have demonstrated that MIF can bind to and activate ACKR3 via both autocrine and paracrine mechanisms (59). Furthermore, the MIF/ACKR3 axis is critical in various biological processes, including lymphocyte chemotaxis and tumor cell proliferation (60). Our study suggests that Tregs may inhibit the activation of ACKR3 through MIF, thereby reducing the inflammatory response of SMCs during the progression of AS. Additionally, CD74 is upregulated during EC injury and contributes to AS progression via the Nuclear factor-k-gene binding (NF-kB) and MAPK signaling pathways (56). MIF interacts with CD74 through a high-affinity binding, and CD74 expression is essential for MIF-mediated ERK-1/2 phosphorylation, which promotes inflammation (61). During AS progression, Tregs may mitigate ERK phosphorylation by reducing MIF levels and their interaction with endothelial CD74, thus decreasing endothelial inflammation. These findings suggest that Tregs may help inhibit AS by regulating inflammation in endothelial and vascular SMCs via the MIF signaling pathway.

To demonstrate the reliability of our dataset analysis results, we constructed AS model using Ldlr^−/−^ mice and investigated the expression of these five transcription factors in mice atherosclerotic plaque. We found that IRF8 and TFEC were also upregulated in mice atherosclerotic plaque. These results indicated that our analysis results are highly reliable, and the main transcription factors could be validated in mice. According to the results of the expression of five transcription factors in human plaque and non-plaque as well as early and advanced plaque, ZNF267, TFEC, and IRF8 were significantly upregulated in atherosclerotic plaque and advanced plaque. ZNF267 has not been validated in atherosclerotic plaque using Ldlr^−/−^ mice. We speculated that there may be different mechanisms of ZNF267 in humans and mice. In previous studies, it has been reported that there are some differences between human and mouse immune systems such as immune cell subsets, receptor expression patterns, and immune response mechanisms (62, 63). In Tregs, CD25 is one of the different identification markers for Tregs identified in mice. However, the use of CD25 expression as a marker is limited in human studies. To mitigate these discrepancies, future studies could validate using primary cells or tissues from human patients and employ larger animal models, such as miniature pigs, that are more consistent with human immune responses.

## Conclusion

This study provides a comprehensive view of the genes and pathways that affect the number and the function of Tregs during the progression of AS plaque. However, our study still has several limitations. Firstly, the data used in this study are based on the GEO database, which contains limited datasets. There may be unaccounted for confounding factors, such as age, sex, BMI, or treatment history, which may lead to bias of the results. Secondly, the prediction model trained in limited datasets may overfit, resulting in inflated performance metrics on the same dataset but poor generalization to new data. The stability of the prediction model also requires more clinical data to be validated. Thirdly, the robust biomarkers identified in the current study were not systematically validated in animals *in vivo* or humans. In future studies, we plan to expand dataset diversity and scope, adjust for additional potential confounding factors, and conduct systematic validation in biological models. By adopting these strategies, future research can address the current study’s limitations, improve the robustness of findings, and enhance the translational potential of the results. In conclusion, our study further confirmed that the proportion of Tregs significantly decreased during atherosclerotic plaque progression. We also identified novel and potential biomarkers, including TFEC, IRF8, KLF2, JUNB, and ZNF267 for Tregs driving the progression of AS. Moreover, ligand–receptor pairs such as the MIF signaling pathway are identified as potential bridges of Tregs to modulate ECs and SMCs function to counteract AS. Our study could be provide potential targets for future predictive diagnostics, patient stratification, targeted prevention, and personalization of medical services in AS.

## Data Availability

The datasets presented in this study can be found in online repositories. The names of the repository/repositories and accession number(s) can be found in the article/**Supplementary Material**.

## Supplementary material

Supplementary Figure 1 The number of 22 subpopulations of immune cells during the progression of carotid atherosclerotic plaque. **(A)** The relative percentage of 22 subpopulations of immune cells in 64 samples was based on the dataset of GSE43292. **(B)** Boxplot of the proportion of 22 subpopulations of immune cells from non-plaque to plaque tissue.

Supplementary Figure 2 The transcription factors of differentially expressed genes in Tregs between PA and AC stage using transcription factor enrichment analysis by orthogonal omics integration. **(A)** The top 10 transcription factors of downregulated genes during the progression of atherosclerotic plaque. **(B)** The top 10 transcription factors of upregulated genes during atherosclerotic plaque progression.

Supplementary Table 1 The personalized signature matrix as the input matrix of reference gene expression signatures.

Supplementary Table 2 The GO-BP enrichment analyses of the 209 key genes.

Supplementary Table 3 The transcription factors of 209 key genes using transcription factor enrichment analysis by orthogonal omics integration.

Supplementary Table 4 The GO-BP enrichment analyses of the 120 significantly differential expressed genes.

Supplementary Table 5 The gene set enrichment analysis (GSEA) of the 120 significantly differential expressed genes.

Supplementary Table 6 The key genes were identified using hdWGCNA in Tregs.
