# Supplementary figures and images for: Integrative single-cell and bulk RNA-seq analyses identify CD4+ T-cell subpopulation infiltration and biomarkers of regulatory T cells involved in mediating the progression of atherosclerotic plaque
Source: Front Immunol. 2025 Jan 17;15:1528475. doi: 10.3389/fimmu.2024.1528475 (PMC11781991; doi:10.3389/fimmu.2024.1528475)

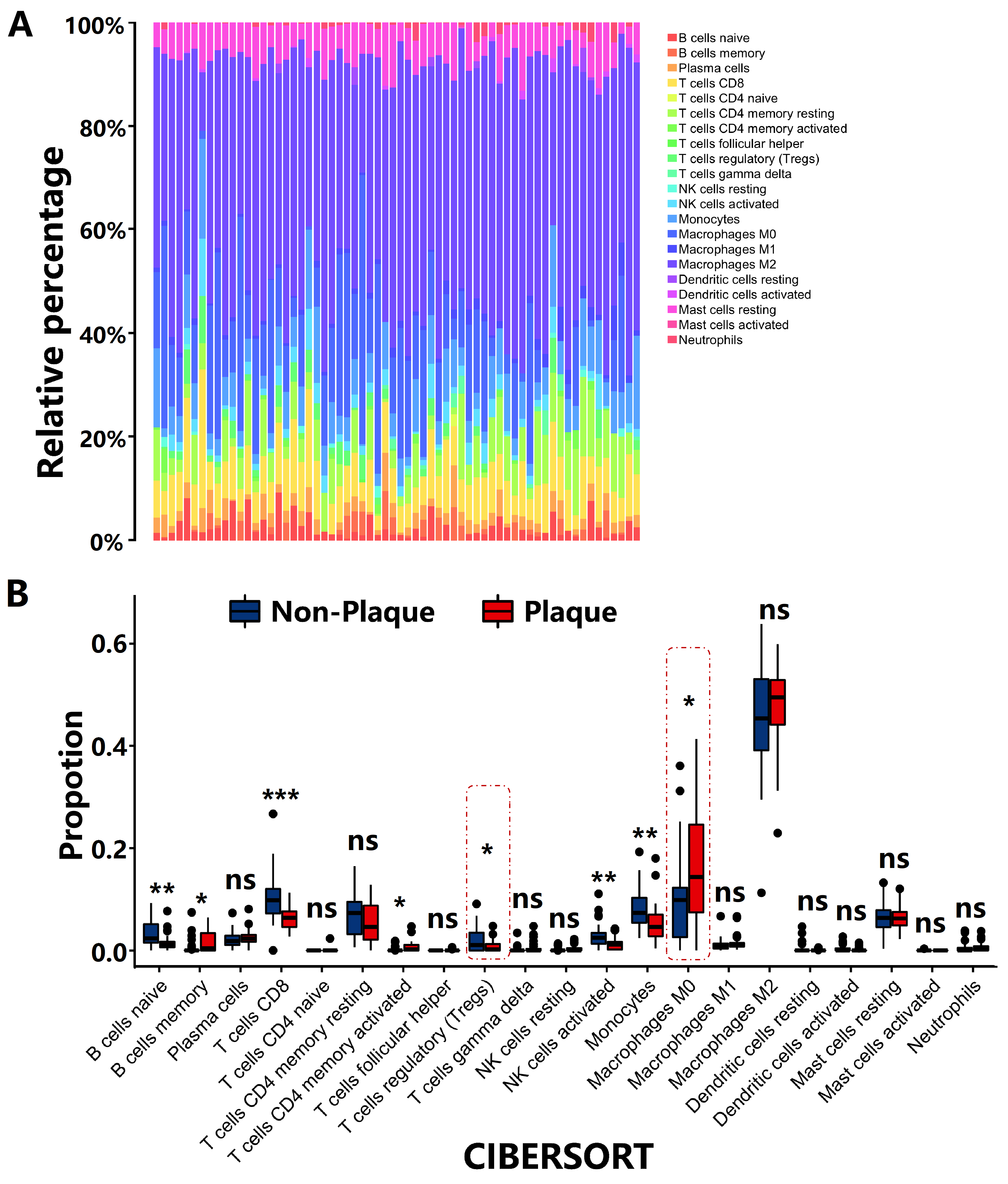

Supplement: Supplementary Figure 1 — The number of 22 subpopulations of immune cells during the progression of carotid atherosclerotic plaque. (A) The relative percentage of 22 subpopulations of immune cells in 64 samples was based on the dataset of GSE43292. (B) Boxplot of the proportion of 22 subpopulations of immune cells from non-plaque to plaque tissue. [file Image1.tif]

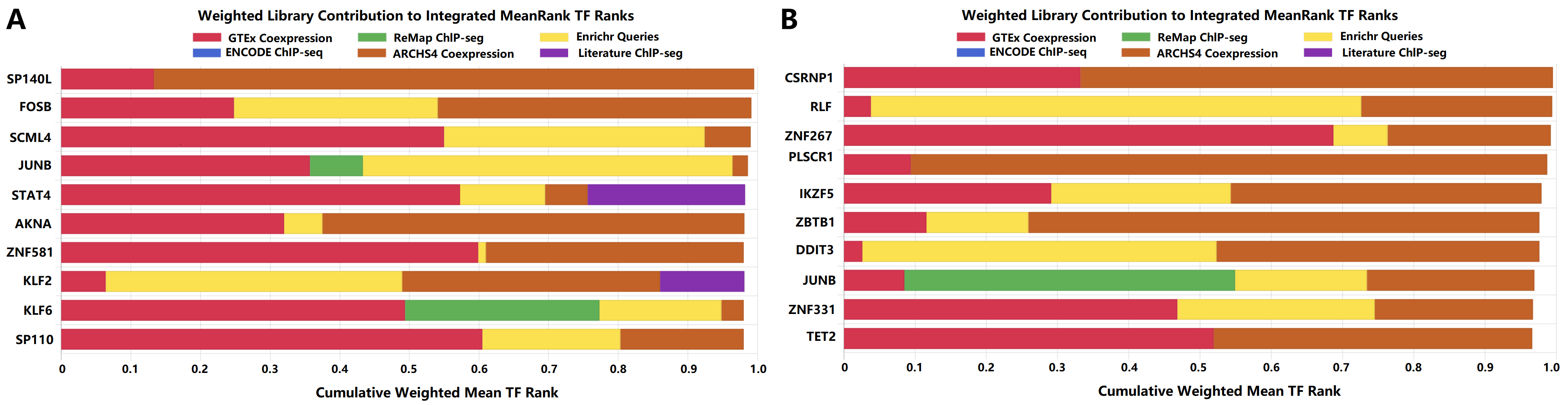

Supplement: Supplementary Figure 2 — The transcription factors of differentially expressed genes in Tregs between PA and AC stage using transcription factor enrichment analysis by orthogonal omics integration. (A) The top 10 transcription factors of downregulated genes during the progression of atherosclerotic plaque. (B) The top 10 transcription factors of upregulated genes during atherosclerotic plaque progression. [file Image2.tif]
